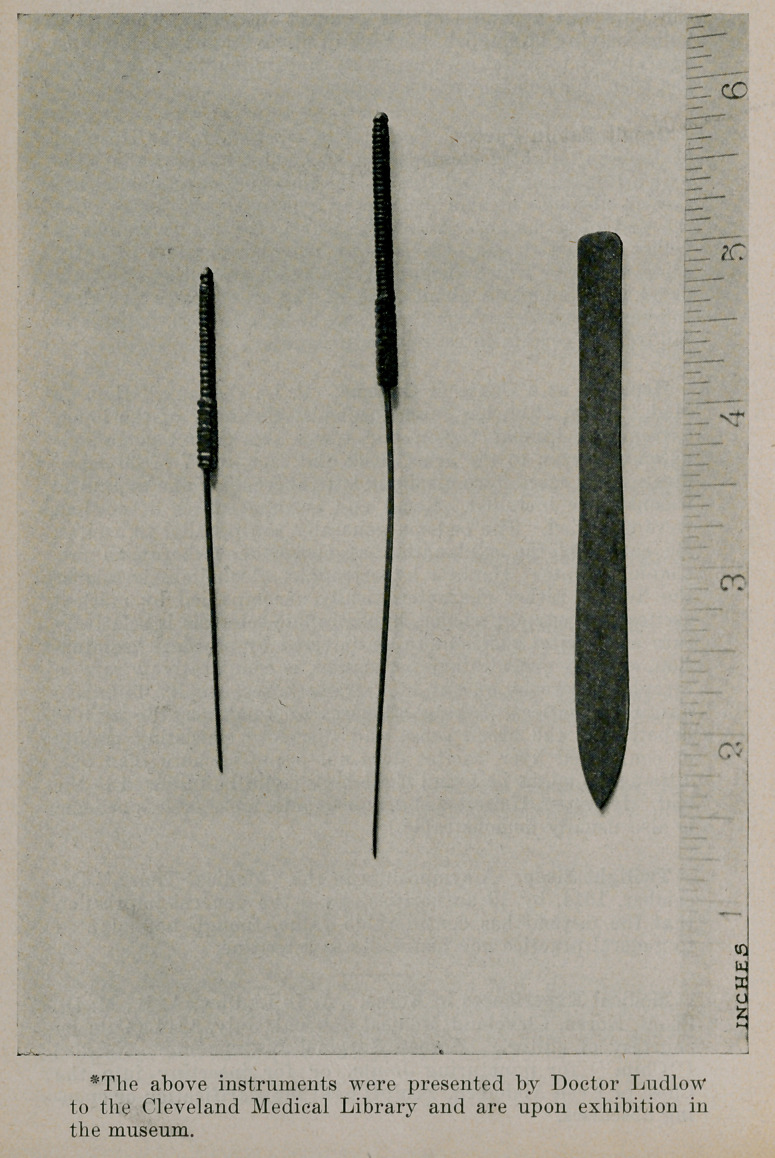# Medical Experiences in Korea

**Published:** 1915-01

**Authors:** 


**Medical Experiences in Korea.** A. L. Ludlow, A. B., M. D., Seoul, Korea, Cleveland Medical Journal, July, 1914 (Cuts by courtesy of editor.) **Korean Surgical Instruments*** Needles ("Chim") for puncturing Joints, or for inserting into the Chest or Even Abdomen. Scalpel used for scarification or opening abscesses.

*The above instruments were presented by Doctor Ludlow to the Cleveland Medical Library and are upon exhibition in the museum.

## Figures and Tables

**Figure f1:**